# Impact of Air Transport on SpO_2_/FiO_2_ among Critical COVID-19 Patients during the First Pandemic Wave in France

**DOI:** 10.3390/jcm10225223

**Published:** 2021-11-09

**Authors:** Jean-Baptiste Bouillon-Minois, Vincent Roux, Matthieu Jabaudon, Mara Flannery, Jonathan Duchenne, Maxime Dumesnil, Morgane Paillard-Turenne, Paul-Henri Gendre, Kévin Grapin, Benjamin Rieu, Frédéric Dutheil, Carolyne Croizier, Jeannot Schmidt, Bruno Pereira

**Affiliations:** 1 Emergency Department, CHU Clermont-Ferrand, F-63000 Clermont-Ferrand, France; vroux@chu-clermontferrand.fr (V.R.); mdumesnil@chu-clermontferrand.fr (M.D.); mpailliard-turenne@chu-clermontferrand.fr (M.P.-T.); phgendre@chu-clermontferrand.fr (P.-H.G.); jschmidt@chu-clermontferrand.fr (J.S.); 2 CNRS, LaPSCo, Physiological and Psychosocial Stress, Université Clermont Auvergne, F-63000 Clermont-Ferrand, France; fdutheil@chu-clermontferrand.fr; 3 Department of Perioperative Medicine, CHU Clermont-Ferrand, F-63000 Clermont-Ferrand, France; mjabaudon@chu-clermontferrand.fr (M.J.); brieu@chu-clermontferrand.fr (B.R.); 4 GReD, CNRS, INSERM, Université Clermont Auvergne, F-63000 Clermont-Ferrand, France; 5 Ronald O. Perelman Department of Emergency Medicine, NYU School of Medicine, New York University Langone Health, New York, NY 10016, USA; Mara.Flannery@nyulangone.org; 6 Emergency Department, CH Aurillac, F-15000 Aurillac, France; j.duchenne@ch-aurillac.fr; 7 Medical Intensive Care Unit, CHU Clermont-Ferrand, F-63000 Clermont-Ferrand, France; kgrapin@chu-clermontferrand.fr; 8 Occupational and Environmental Medicine, CHU Clermont-Ferrand, WittyFit, F-63000 Clermont-Ferrand, France; 9 Hematology Department, CHU Clermont-Ferrand, F-63000 Clermont-Ferrand, France; ccroizier@chu-clermontferrand.fr; 10 Clinical Research and Innovation Direction, CHU Clermont-Ferrand, F-63000 Clermont-Ferrand, France; bpereira@chu-clermontferrand.fr

**Keywords:** COVID, emergency medicine, public health, air transport, ARDS

## Abstract

During the first wave of the COVID-19 pandemic, some French regions were more affected than others. To relieve those areas most affected, the French government organized transfers of critical patients, notably by plane or helicopter. Our objective was to investigate the impact of such transfers on the pulse oximetric saturation (SpO_2_)-to-inspired fraction of oxygen (FiO_2_) ratio among transferred critical patients with COVID-19. We conducted a retrospective study on medical and paramedical records. The primary endpoint was the change in SpO_2_/FiO_2_ during transfers. Thirty-eight patients were transferred between 28 March and 5 April 2020, with a mean age of 62.4 years and a mean body mass index of 29.8 kg/m^2^. The population was 69.7% male, and the leading medical history was hypertension (42.1%), diabetes (34.2%), and dyslipidemia (18.4%). Of 28 patients with full data, we found a decrease of 28.9 points in SpO_2_/FiO_2_ (95% confidence interval, 5.8 to 52.1, *p* = 0.01) between the starting and the arrival intensive care units (SpO_2_/FiO_2_, 187.3 ± 61.3 and 158.4 ± 62.8 mmHg, respectively). Air medical transfers organized to relieve intensive care unit teams under surging conditions during the first COVID wave were associated with significant decreases in arterial oxygenation.

## 1. Introduction

Since the description of the new coronavirus disease 2019 (COVID-19) [1] caused by the severe acute respiratory syndrome coronavirus 2 (SARS-CoV-2), a major challenge for governments worldwide has been to continuously adapt their healthcare policies to the viral spread, availabilities of equipment and preventive or therapeutic measures, and medico–economic considerations [2]. The most severe manifestation of COVID-19 is acute respiratory distress and impaired arterial oxygenation requiring oxygen therapy and/or mechanical ventilation [3]. When the COVID-19 pandemic started, worldwide experts recommended tracheal intubation in the presence of criteria for acute respiratory distress syndrome (ARDS) [4]. Diagnosis and prognostic criteria for ARDS are those from the Berlin definition, including a ratio of the partial pressure of arterial oxygen (PaO_2_) to the fraction of inspired oxygen (FiO_2_) of 300 mmHg or less [5]. This criterion requires an invasive measurement to obtain PaO_2_ and is mainly available in the hospital. The pulse oximetric saturation (SpO_2_)-to-FiO_2_ ratio has been proposed as a reliable surrogate for the PaO_2_/FiO_2_ ratio in patients with ARDS and could be a readily available tool for monitoring critical patients during medical transfers [6,7].

The localized epidemic rapidly became a global pandemic, and governments worldwide were forced to impose a generalized lockdown [8]. At its peak, more than half of the world’s population was confined [9]. In France, this lockdown was declared on 17 March 2020. However, some French regions, such as the Grand-Est and Paris, were overcrowded with critically ill patients [10]. Despite the rapid creation of ephemeral intensive care units (ICUs), intensive care beds were lacking, and healthcare workers from these regions were exhausted and experiencing burnout [11]. In other regions such as Auvergne, the lockdown took place before the virus circulation was very active. Admissions to emergency departments (EDs) were halved [12]. This, in tandem with cancellation of all non-urgent surgical procedures and medical consultations, induced a record number of available beds [13]. Therefore, the French government, with the support of the French Society of Emergency Medicine (SFMU) and SAMU-Urgences de France (SUDF), decided to transfer ICU patients from overcrowded regions to regions where beds were available. Medical transport was carried out by mobile ICU teams composed of emergency physicians (EPs) and state-qualified nurses, in partnership with military units. This large-scale civil–military cooperation was unprecedented in the history of the French healthcare system. Several means of transport were used, including road (resuscitation ambulances), rail (high-speed trains), and air (civil and military aircraft or helicopters).

The main objective of our study was to examine the changes in the SpO_2_/FiO_2_ ratio between the start of transfer and arrival at ICUs in patients transferred to the Auvergne region during the first wave of the COVID-19 pandemic. Secondary objectives were to assess changes in other vital parameters (such as arterial blood pressure or heart rate) and to identify other factors influencing the evolution of the ventilatory needs of transferred patients.

## 2. Materials and Methods

### 2.1. Air Medical Transport

**Figure 1:**
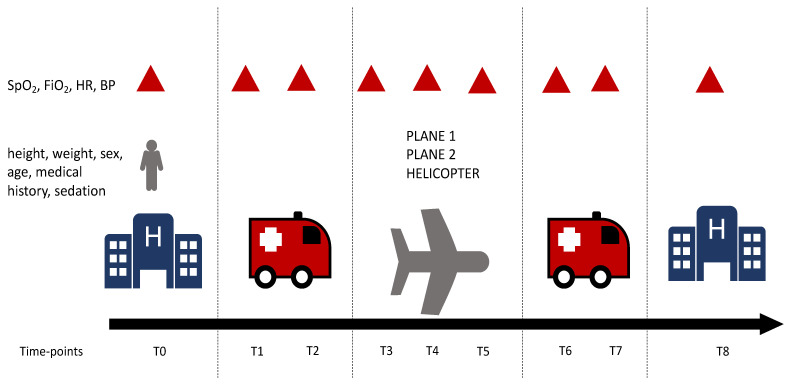
Patient’s journey. SpO_2_ = pulse oxygen saturation, FiO_2_ = inspired fraction of oxygen; HR = heart rate; BP = invasive blood pressure; T0 = before leaving the starting ICU, T1 = at the beginning of the transport between the starting ICU and the airport or drop zone (DZ), T2 = at the end of the transportation between the starting ICU and the airport or DZ, T3 = at the beginning of the flight, T4 = in the middle of the flight, T5 = at the end of the flight, T6 = at the beginning of the transport between the airport or DZ and the arrival ICU, T7 = at the end of the transportation between the airport or DZ and the arrival ICU and T8 = arrival in the ICU.

We performed a retrospective multicenter study comprising patients with severe COVID-19, hospitalized in thirteen ICUs in regions under surging conditions and transferred to ICUs in hospitals in the Auvergne region (low incidence of COVID-19) between 28 March 2020 and 5 April 2020. The transfer between ICUs took place in three stages—first from the starting ICU to the airport or the helicopter’s dropping zone (DZ); second, between the departure airport or DZ and the arrival airport or DZ; third, between the airport or arrival DZ and the arrival ICU. During the entire journey, patients were intubated and ventilated with Monnal T60^®^ (Air Liquide Healthcare, Paris, France) transport respirators and monitored with a scope including EKG, invasive blood pressure, and pulse oximetric saturation. Each patient was cared for by at least one emergency physician (EP) and one certified nurse. During the transport, all healthcare providers were dressed in Tyvek^®^-type personal protective equipment (Dupont, Wilmington, DE, USA) protective goggles, and a filtering facepiece type 2 (FFP2) or N95 facial mask [14]. During each transfer, another EP, considered as the “leader”, supported the transfer teams and allocated patients to the different team members. Patients could be transported with Airbus H225M helicopters (Airbus, Blagnac, Toulouse, France) with a maximal capacity of three patients, with Piaggio P180 Avanti planes (Piago, Genova, Italy) with a maximal capacity of two patients, or with a military Airbus A400M Atlas plane (Airbus, Blagnac, Toulouse, France) with a maximal capacity of five patients (Figure 1).

### 2.2. Data Collection

Data were retrospectively collected from medical and nursing records during the whole transport (starting ICU, mobile ICUs, and arrival ICU). Data included the location of the starting ICU, location of the arrival ICU, the duration of transport, the type of aircraft or helicopter, the type of sedative drugs, the length of stay in the arrival ICU, mortality at 7 and 30 days, socio-demographics (sex, age, height, weight, body mass index), and history of diabetes, arterial hypertension, dyslipidemia, or neoplasia. In addition, vital parameters, defined as SpO_2_, invasive systolic blood pressure (SBP), invasive diastolic blood pressure (DBP), invasive mean blood pressure (MBP), and heart rate (HR) were collected at each time point. For a description of time points, see Figure 1.

### 2.3. Data Analysis

Study data were compiled in an Excel spreadsheet before statistical analyses were performed with Stata^®^ 16 (StataCorp, College Station, TX, USA). A two-sided *p* value of less than 0.05 was considered for statistical significance.

The primary analysis focused on the change in SpO_2_/FiO_2_ (=delta SpO_2_/FiO_2_) between the starting ICU and the arrival ICU (primary outcome), as defined as ((SpO_2_/FiO_2_)a-(SpO_2_/FiO_2_)b)/(SpO_2_/FiO_2_)a, where “a” represents the starting ICU and “b” the arrival ICU.

Patient characteristics were expressed as numbers and percentages for categorical variables and as means ± standard deviations (SD) for quantitative variables, according to the statistical distribution. The assumption of normality was assessed by the Shapiro–Wilk test.

The comparisons between independent groups (such as gender, hypertension, and diabetes) for continuous variables were performed using non-paired t-test for Gaussian variables and Kruskal–Wallis test if not. For categorical variables, the comparisons were performed with the chi-squared or Fisher’s exact tests. Relationships between transfer continuous parameters, including SpO_2_/FiO_2_ and its change, were assessed using Spearman correlation and were interpreted as follows: (0; 0.2) or (−0.2; 0): very weak; (0.2–0.5) or (−0.5; −0.2): weak to mild; up to 0.5 (less than −0.5) strong to very strong.

Furthermore, a mixed model for repeated data was computed to analyze the change in SpO_2_/FiO_2_ during the different steps of the transfer, considering time as fixed effect and patient as random effect to model between-subjects and within-subjects variabilities. A Sidak’s type I error correction was applied for multiple comparisons between time points evaluation.

Particular attention was paid to missing data. First, a sensitivity analysis was conducted on complete cases, i.e., for patients without missing data for the primary outcome. Furthermore, patients with and without missing data (for the primary outcome) were compared for their main characteristics to evaluate the sample representativeness. The results are expressed using Hedges’ effect-size.

### 2.4. Ethics

An ethical agreement was requested and obtained on 15 July 2020 from the Institutional Review Board of the Clermont-Ferrand University Hospital (CPP Sud-Est VI) under the reference 2020/CE 51.

## 3. Results

### 3.1. Study Population

A total of 38 patients were included. All sociodemographic criteria are grouped in Table 1. The average age was 62.4 years ± 11.

The average body mass index was 29.8 kg/m^2^ ± 5.9. The population comprised 25 males (65.8%) and 13 females (34.2%) for a sex ratio = 2.1. A total of 13 patients (34.2%) had a history of diabetes, 16 (42.1%) of hypertension, and 7 (18.4%) of dyslipidemia. We did not have any patient on vasopressors. There were 6 patients (15.8%) who were sedated with propofol, 18 (47.4%) with midazolam, 9 (23.7%) with both propofol and midazolam. Sufentanil was the analgesic for 31 patients (81.6%), remifentanil for 2 (5.3%). Before leaving the initial ICU, mean vital signs were as follows: systolic blood pressure was 124 mmHg ± 29, diastolic blood pressure was 67 mmHg ± 21, mean blood pressure was 86 mmHg ± 22, heart rate was 80 pulses/min ± 14, SpO_2_ was 95% ± 4, and FiO_2_ was 58% ± 21.

Mean SpO_2_/FiO_2_ was 187.3 ± 61.3 (Table 1). All patients were ventilated using an assist-control mode. We do not have any data about respiratory rate, tidal volume, or positive end expiratory pressure. Before leaving their starting ICU, 3 patients had a SpO_2_/FiO_2_ of 100 or less, 14 between 101 and 200, 10 between 201 and 300, and 1 up to 301. Data on SpO_2_/FiO_2_ in starting ICU were missing for ten patients. There were 11 patients (29.7%) who were transported with an A400m military plane, 11 (29.7%) with a civil plane, and 15 (40.5%) with Airbus H225M helicopters.

The mean duration of hospitalization in the arrival ICU was 22.8 days ± 17.4. One patient (2.6%) died during the first 7 days and nine (23.7%) during the first 30 days. Using Fisher’s exact test, we compared baseline characteristics between patients with available data on the primary outcome (i.e., SpO_2_/FiO_2_ measurement in all different times) and those without, and no difference was found (Appendix A, Table A1).

### 3.2. Primary Outcome

**Figure 2:**
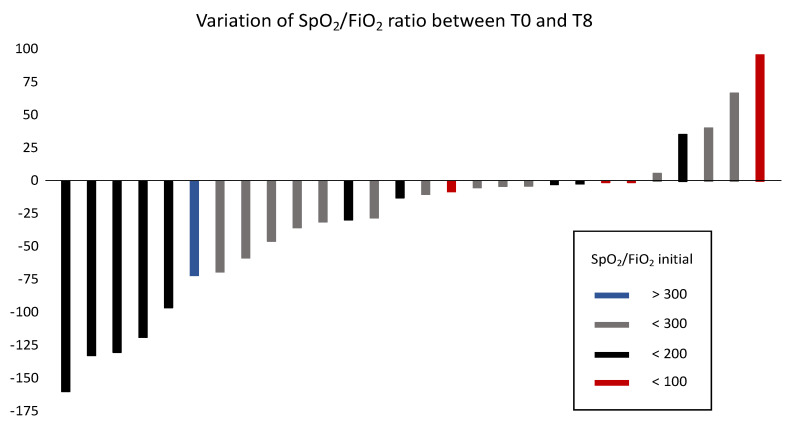
Change in SpO_2_/FiO_2_ between the starting and arrival intensive care units among 28 transferred patients.

SpO_2_/FiO_2_ data from both the starting and the arrival ICUs were available for 28 patients. The mean change in SpO_2_/FiO_2_ between the starting and the arrival ICUs was a decrease of −28.9 points (95% confidence interval, −52.1 to −5.8; −11.7%, *p* = 0.016) between the initial and final SpO_2_/FiO_2_ values. SpO_2_/FiO_2_ increased during transport in 5 patients, with a maximal increase of +96 points (+104%) in 1 patient, and it decreased in 23 patients, with a maximal decrease of −160 points (−60%) in one patient (Figure 2).

### 3.3. Factors Associated with Changes in SpO_2_/FiO_2_ during Transport

There was no significant relationship between the change in SpO_2_/FiO_2_ during transport and patient’s sex (*p* = 0.446), a history of diabetes (*p* = 0.945) or dyslipidemia (*p* = 0.529), and with type of air transport (helicopter or aircraft) (*p* = 0.729). Patients with a personal history of hypertension had a significant increase in SpO_2_/FiO_2_ during transport, compared with those who did not (*p* = 0.019). There was no correlation between changes in SpO_2_/FiO_2_ during transport and age (*p* = 0.295), height (*p* = 0.771), weight (0.479), body mass index (*p* = 0.469), or with vital parameters measured at the starting ICU, such as heart rate (*p* = 0.470), systolic blood pressure (*p* = 0.530), diastolic blood pressure (*p* = 0.935), mean blood pressure (*p* = 0.739), or the initial SpO_2_/FiO_2_ (*p* = 0.240). However, there was a significant correlation between the duration of the transport and changes in SpO_2_/FiO_2_ during the transport (correlation coefficient = −0.425, *p* = 0.048).

### 3.4. Evolution of SpO_2_/FiO_2_ during the Transport

**Figure 3:**
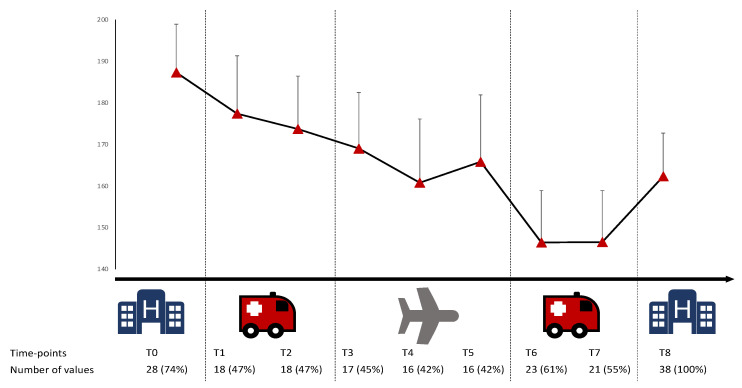
Evolution of SpO_2_/FiO_2_ during transport + standard error of the mean. T0 = before leaving the starting ICU, T1 = at the beginning of the transport between the starting ICU and airport or drop zone (DZ), T2 = at the end of the transportation between the starting ICU and airport or DZ, T3 = at the beginning of the flight, T4 = in the middle of the flight, T5 = at the end of the flight, T6 = at the beginning of the transport between the airport or DZ and the arrival ICU, T7 = at the end of the transportation between the airport or DZ and the arrival ICU and T8 = the arrival in the final ICU.

Figure 3 summarizes changes in SpO_2_/FiO_2_ during all phases of the transport. Although there was an overall decrease in SpO_2_/FiO_2_ during transport, the decrease is most important between landing and the beginning of the transport between the airport or DZ and the arrival ICU, before SpO_2_/FiO_2_ increases again upon arrival at the final ICU. Using a mixed method analysis incorporating time effect and Sidak’s correction, we studied the changes in the SpO_2_/FiO_2_ ratio during the transport, considering the initial SpO_2_/FiO_2_ (as measured when leaving the starting ICU) as the reference. The decrease in SpO_2_/FiO_2_ began during the transfer between the starting ICU and the airport or DZ (−17.8 points; 95% CI, −37.0 to −1.4), and the decrease was maximal after landing (−52.6 points; 95% CI, −69.3 to −35.9). In contrast, there was a relative increase in SpO_2_/FiO_2_ between the airport or DZ and arrival in the ICU (+27.4 points; 95% CI, 10.7 to 43.9). However, the final SpO_2_/FiO_2_ (as measured upon admission to the arrival ICU) was lower than the initial SpO_2_/FiO_2_ (−27.7 points; 95% CI, −42.2 to −13.1).

When restricting analyses to the twelve patients with full data (SpO_2_/FiO_2_ at each time point) available, there was a significant decrease in SpO_2_/FiO_2_ between the end of the flight and the beginning of the transport from the airport or DZ to the arrival ICU (−24.6 point; 95% CI, 0.79 to 48.5, *p* = 0.04). We also found an increase in SpO_2_/FiO_2_ between the beginning of the transport from the airport or DZ and the arrival ICU (+27.4 point; 95% CI, 10.7 to 44, *p* = 0.002). Using a mixed method analysis and Sidak’s correction, we studied the evolution of SpO_2_/FiO_2_ ratio during the journey, using initial SpO_2_/FiO_2_ as baseline for comparison. Very interestingly, the decrease in SpO_2_/FiO_2_ began during the transfer between initial ICU and airport/DZ (−17.8; 95% Confidence Interval −37 to −1.4). We also found a peak of decrease after landing (−52.5, 95% CI −69.3 to −35.9). Finally, we found a relative increase in SpO_2_/FiO_2_ between airport/DZ and arrival in the ICU. However, final SpO_2_/FiO_2_ was not back to initial ratio but to mid-flight value (−27.7, 95% CI −42.2 to −13.1).

## 4. Discussion

To our knowledge, our study is the first to report a negative impact of air transport on oxygenation, as assessed by the SpO_2_/FiO_2_ ratio, in critical patients with COVID-19. Furthermore, we found a negative impact of duration of transport, especially during landing and switch of teams, but the decrease was stabilized as soon as ground transport between DZ and arrival ICU was initiated.

### 4.1. ARDS in COVID-19

ARDS is a severe form of acute inflammatory lung injury and alveolar edema. It is associated with a high level of mortality and significant morbidity. Since COVID-19 affects the respiratory system, some patients are fast progressing to ARDS. However, there are many differences between COVID-19-related ARDS and ARDS caused by other factors. Berlin criteria are considered the gold standard for ARDS diagnosis, but COVID-19 ARDS does not respond to all criteria. Indeed, COVID-19 ARDS is not responding to the one-week delay, and lung compliance can be relatively high in COVID-19-related ARDS patients. Thus, authors proposed different phenotypes of COVID-19 ARDS: Type 1 with high pulmonary compliance (>50 mL/cmH_2_O), low lung elastance, low lung weight, and low lung recruitability and type 2 with markedly reduced pulmonary compliance (<40 mL/cmH_2_O), high lung elastance, high lung weight (>1.5 kg), and high lung recruitability [15]. Type 1 patients are also named “happy hypoxemia”. Those patients did not feel uncomfortable despite a low PaO_2_/FiO_2_, CT scans abnormalities, and a need for oxygen therapy [16]. Arterial hypoxemia COVID-19 is caused by a mismatch between ventilated versus perfused lung areas and thus persistence of pulmonary arterial blood flow to the non-ventilated area [3].

### 4.2. The Link between PaO_2_/FiO_2_ and SpO_2_/FiO_2_

Berlin criteria are based on the PaO_2_/FiO_2_ ratio, which requires an invasive measure of arterial blood gas and is not available for continuous follow-up. The link between PaO_2_ and SpO_2_ is sinusoidal. However, many studies investigating the connection between these two values used a linear or log-linear regression model. A single retrospective study in ICU ARDS patients established that a nonlinear equation more accurately imputes PaO_2_/FiO_2_ from SpO_2_/FiO_2_ versus linear or log-linear equations, with similarly observed hospital mortality as a function of SpO_2_/FiO_2_ ratio compared with measured PaO_2_/FiO_2_ ratios [17]. Furthermore, SpO_2_ in COVID-19 must be interpreted with caution. Indeed, COVID-19 induces tachypnea, which leads to hypocapnia and therefore alkalosis. This alkalosis shifts the oxyhemoglobin dissociation curve to the left, explaining why SpO_2_ is still preserved in cases with a low PaO_2_ (3). Another hypothesis concerns the impact of SARS-CoV-2 on the heme group of hemoglobin. Heme serum levels are increasing in COVID-19 and harmful iron ions (Fe^3+^), leading to inflammation and therefore the production of large amounts of serum ferritin to bind these free irons to reduce tissue damage [18].

### 4.3. The Necessity to Help Overcrowded Areas

During the first wave of the COVID-19 pandemic, some French regions were overcrowded, such as Paris and the Grand-Est region [19]. However, in others such as Auvergne, hospitals were empty because the lockdown was active before the virus circulated with a high incidence [12,13]. As a result, EDs showed a considerable decrease in consultations. Furthermore, all non-urgent surgeries and medical exams were canceled, resulting in many more beds available than patients who required admission. Lastly, in all hospitals in France, temporary ICUs were created to accept a tsunami of patients. In those ICUs, and because of the lack of available ventilators, transport ventilators were used. Although those transport ventilators can be used safely, they are still a temporary measure. Indeed, it is not possible to precisely titrate FiO_2_ on a point-by-point basis but only to adjust per five percent increase or decrease. In addition, adjustments and monitoring of the I/E ratio are different from, and far less precise than, those for an ICU ventilator [20]. At the end of March 2020, the French government had to choose between two options: either organize the transfer of ICU workers from empty regions to overcrowded regions, or transfer patients already in ICUs. At the end of March 2020, nobody knew if empty regions would experience delayed overcrowding, so the government did not risk transferring emergency and ICU healthcare workers to empty regions. Considering that France has had a vast and robust out-of-hospital experience with EPs in ambulances for decades to transport very ill patients, the French government decided to transport patients to decrease the workload on ICU workers [21]. Considering that even intra-hospital transport can induce more than 67% of unexpected events, mainly represented by SpO_2_ issues, a transport of a severe COVID-19 patient between two hospitals, using two ambulances and one air transport, requires robust coordination to ensure the best patient care It requires training, good logistics and coordination to decrease the incidence of serious adverse events. Transport teams were all composed of at least one EP and one emergency nurse for each patient, and another physician was present as a team leader to coordinate the transport, but also as a referent in case of adverse event or need. However, a previous study showed that short air transport (25 min) for COVID-19 patients can be safe and feasible [22,23].

### 4.4. Impact of Landing and Team Switch

In our study, the SpO_2_/FiO_2_ ratio decreased by 43.6 points during the landing/team switch, compared with the initial ICU (*p* = 0.03), and by 29.4 points between the flight and the landing/team switch, but it gradually rose again during the short transfer between the airport or DZ and the arrival ICU. These data are interesting, but we have not found a clear explanation in the literature. The use of digital SpO_2_ monitoring can induce a high incidence of signal latency associated with desaturations during prehospital, especially during landing and team switches [24]. However, pre-hospital teams are aware of this issue and how to manage it. Although helicopter or ambulance movement was the main cause of acute issues in SpO_2_ monitoring, vasoconstriction was the main cause of bad SpO_2_ signal. However, we did not have any patients on vasopressors, which is in accordance with the literature [25]. Indeed, it is known that high altitudes, with the decrease in barometric pressures, decrease SpO_2_. However, even if the planes or helicopters used during these transfers were not fully pressurized, they did not fly up to 5000 feet, which is insufficient to explain these variations. Another hypothesis lies in the sudden change in altitude that could potentially explain these variations, but we did not find any explanation in the literature. A third hypothesis, linked to the second, is the short deceleration time. The last hypothesis is the change of team and material. Indeed, in March 2020, recommendations to change respiratory materials were to clamp the intubation tube before switching respiratory materials to decrease the spread of the virus. This induced a short period (between 15 and 30 s) of apnea. However, the rapid recovery prompted us to think that all those causes may have been involved in this degradation. Indeed, we found an increase in pulmonary function during the transport between DZ and ICU arrival. Another explanation is the low monitoring abilities of transport respirators. Furthermore, although emergency physicians and emergency nurses are fully capable of managing respiratory distress and ARSD in the first contact with patients, they were not specialists on mechanical ventilation, especially on COVID ventilation at the time of the first wave. Lastly, the extreme fragility of the patients and the lack of reserve of lung capacity may be a factor of distress.

### 4.5. Limitations

First, our cohort comprised only 38 patients, only 28 had a SpO_2_/FiO_2_ value in the starting ICU, and only 12 had complete data, which limits the power of our results. This is part of the disadvantage of using retrospective data from a cohort study. Indeed, the data available for this type of study may be absent or of poor quality. Potential confounding factors may have been missed. However, with 198 measurements, we found an essential impact of air transport. Moreover, the lack of a control arm (another issue of retrospective cohort studies) did not allow us to conclude a specific effect of air transport on SpO_2_/FiO_2_ ratio. However, despite the low number of patients, we were able to observe a significant change in patients with complete data and during landing and change on teams. These preliminary results need to be confirmed in a larger population and in a randomized controlled study to compare different types of transport (air, rail, road). Another limitation is our inability to assess the impact of changes in ventilator settings (respiration rate, positive end-expiratory pressure, tidal volume) during the transfer. Unfortunately, we did not have enough data on muscle relaxation therapy use and on ventilation parameters during different times of transport. It could be relevant to have those data to more precisely study the impact of those two variables on oxygenation. Last, it could also be interesting to conduct a long-term follow-up of those patients. Indeed, we do not have any data regarding long-term morbidity and mortality among our patients. However, we did not have any deaths during the transport.

## 5. Conclusions

During the first wave of the COVID-19 pandemic in March and April 2020, France was experiencing overcrowded (Paris, Grand-Est) and empty (Auvergne) areas. To help overcrowded areas, 33 intubated patients were transferred to ICUs from the Auvergne region and medical transport was performed with helicopters or planes. During such transfers by air, the SpO_2_/FiO_2_ ratio significantly decreased (−28.9 points; 95% CI, −52.1 to −5.8) especially during the landing, team switch, and when duration of transport increased.

## Figures and Tables

**Table 1:** Population characteristics. SD = standard deviation, n = number of patients, % = percentage, cm = centimeters, kg = kilograms, m^2^ = square meters, mmHg = millimeter of mercury, SpO_2_ = oxygen saturation, FiO_2_ = percentage of oxygen.

Characteristics	Mean ± SD *n* (%)
Age (years)	62.4 ± 11.1
Height (cm)	172.2 ± 10
Weight (kg)	88.6 ± 22.1
Body Mass Index (kg/m^2^)	29.8 ± 5.9
Length of hospitalization in intensive care unit (days)	22.8 ± 17.5
Vital signs in initial intensive care unit	
Systolic Blood Pressure (mmHg)	124 ± 29
Diastolic Blood Pressure (mmHg)	67 ± 21
Mean Blood Pressure (mmHg)	86 ± 22
Heart Rate (pulse per minute)	80 ± 14
SpO_2_ (%)	95 ± 4
FiO_2_ (%)	58 ± 21
SpO_2_/FiO_2_	187.3 ± 61.3
Vital signs in arrival intensive care unit	
Systolic Blood Pressure (mmHg)	124 ± 26
Diastolic Blood Pressure (mmHg)	67 ± 13
Mean Blood Pressure (mmHg)	86 ± 15
Heart Rate (pulse per minute)	84 ± 20
SpO_2_ (%)	94 ± 7
FiO_2_ (%)	67 ± 25
SpO_2_/FiO_2_	162.3 ± 64.3
Sex	
Male	25 (65.8)
Female	13 (34.2)
Medical History	
Diabetes	13 (34.2)
Hypertension	16 (42.1)
Dyslipidemia	7 (18.4)
Date of transport	
28 March	10 (26.3)
29 March	8 (21.1)
30 March	1 (2.6)
3 April	6 (15.8)
4 April	12 (31.6)
5 April	1 (2.6)
Type of flight	
A400m	11 (29.7)
Small plane	11 (29.7)
Helicopter	15 (40.5)
Type of sedative	
Midazolam	18 (47.4)
Propofol	6 (15.8)
Midazolam + Propofol	9 (23.7)
Missing Data	5 (13.2)
Type of analgesia	
Remifentanil	2 (5.3)
Sufentanyl	31 (81.6)
Missing Data	5 (13.2)
Mortality	
7 days	1 (2.6)
30 days	9 (23.7)

## Data Availability

All data available can be requested from the corresponding author.

## Appendix A

**Table A1:** Baseline characteristics of patients with data in all nine time points of measurement and those without data in all nine time points of measurement.

Characteristics	Mean ± SD *n* (%) Full Data (*n* = 12)	Mean ± SD *n* (%) Missing Data (*n* = 26)	Hedge’s IgI or Effect Size
Age (years)	60.3 ± 10.5	63.4 ± 11.4	−0.279
Height (cm)	169.2 ± 10.4	173.5 ± 9.7	−0.431
Weight (kg)	82.4 ± 11.8	91.4 ± 25.2	−0.404
Body Mass Index (kg/m^2^)	28.7 ± 3.1	30.3 ± 6.8	−0.262
Vital signs in initial intensive care unit			
Systolic Blood Pressure (mmHg)	126.6 ± 31	118.9 ± 26.8	0.251
Diastolic Blood Pressure (mmHg)	70.4 ± 22	62.3 ± 19.2	0.347
Mean Blood Pressure (mmHg)	89.1 ± 23.7	81.2 ± 18.7	0.373
Heart Rate (pulse per minute)	78.3 ± 15	81.9 ± 13.8	−0.237
SpO_2_ (%)	96.2 ± 3.1	94.1 ± 4	0.539
FiO_2_ (%)	53.3 ± 12.5	60.1 ± 25.6	−0.351
SpO_2_/FiO_2_	192.1 ± 56	183.8 ± 66.6	0.129
Sex			
Male	8 (66.7)	17 (65.4)	0.027
Female	4 (33.3)	9 (34.6)	
Medical History			
Diabetes	2 (16.7)	11 (42.3)	−0.586
Hypertension	4 (33.3)	12 (46.2)	−0.264
Dyslipidemia	0	7 (26.9)	
